# Potential Mechanisms and Perspectives in Ischemic Stroke Treatment Using Stem Cell Therapies

**DOI:** 10.3389/fcell.2021.646927

**Published:** 2021-04-01

**Authors:** Guoyang Zhou, Yongjie Wang, Shiqi Gao, Xiongjie Fu, Yang Cao, Yucong Peng, Jianfeng Zhuang, Junwen Hu, Anwen Shao, Lin Wang

**Affiliations:** Department of Neurosurgery, The Second Affiliated Hospital, School of Medicine, Zhejiang University, Hangzhou, China

**Keywords:** stem cell, cell therapy, ischemic stroke, transplantation, clinical trial, regenerative medicine

## Abstract

Ischemic stroke (IS) remains one of the major causes of death and disability due to the limited ability of central nervous system cells to regenerate and differentiate. Although several advances have been made in stroke therapies in the last decades, there are only a few approaches available to improve IS outcome. In the acute phase of IS, mechanical thrombectomy and the administration of tissue plasminogen activator have been widely used, while aspirin or clopidogrel represents the main therapy used in the subacute or chronic phase. However, in most cases, stroke patients fail to achieve satisfactory functional recovery under the treatments mentioned above. Recently, cell therapy, especially stem cell therapy, has been considered as a novel and potential therapeutic strategy to improve stroke outcome through mechanisms, including cell differentiation, cell replacement, immunomodulation, neural circuit reconstruction, and protective factor release. Different stem cell types, such as mesenchymal stem cells, marrow mononuclear cells, and neural stem cells, have also been considered for stroke therapy. In recent years, many clinical and preclinical studies on cell therapy have been carried out, and numerous results have shown that cell therapy has bright prospects in the treatment of stroke. However, some cell therapy issues are not yet fully understood, such as its optimal parameters including cell type choice, cell doses, and injection routes; therefore, a closer relationship between basic and clinical research is needed. In this review, the role of cell therapy in stroke treatment and its mechanisms was summarized, as well as the function of different stem cell types in stroke treatment and the clinical trials using stem cell therapy to cure stroke, to reveal future insights on stroke-related cell therapy, and to guide further studies.

## Introduction

Stroke is a common cerebrovascular disease with high rates of fatality and disability (Feigin et al., 2017) and is the second leading cause of death, with 5.5 million deaths every year (GBD 2016 Stroke Collaborators, 2019; Lindsay et al., 2019). In the acute phase of ischemic stroke (IS), mechanical thrombectomy (MT), and the administration of tissue plasminogen activator (tPA) can recanalize the occlusive lesion. However, these approaches are limited by the narrow time window for application of 3–4.5 h after IS onset in case of thrombolytic therapy (Roth, 2011; Wardlaw et al., 2014) and up to 24 h after stroke onset in case of MT, depending on imaging criteria (Thomalla and Gerloff, 2019), and only 5–10% of patients are eligible to receive a reperfusion treatment (Mozaffarian et al., 2016). In the subacute to chronic phases, stroke treatment strategies may be changed to long-term antiplatelet or anticoagulation drugs depending on patients’ condition (Bala et al., 2020) or intervention in patients’ risk factors of stroke such as hypertension, diabetes, and hypercholesterolemia (Rundek and Sacco, 2008; Tziomalos et al., 2009). While these treatments show significant benefits in stroke patients, they are still not enough to ensure an acceptable quality of life. Recently, cell therapy has gained much attention as a potential IS treatment, since the results obtained so far suggest it has a bright future. Several types of cell therapy are available, including stem cell therapy (Suda et al., 2020), polarized cell therapy (Jiang et al., 2013), and genetic modification or pretreatment stem cell therapy (Kurozumi et al., 2005; Damasceno et al., 2020). In recent years, stem cell therapy has garnered more attention due to its satisfactory results in some preclinical and clinical studies. We believe that stem cell therapy will make an important contribution to the field of stroke treatment, and further research will compliment and add to the successful results seen when using other cell types. The stem cells used in cell therapy include mesenchymal stem cells (MSCs), neural stem cells (NSCs), induced pluripotent stem cells (iPSCs), hematopoietic stem cells (CD34-positive) (HSCs), dental pulp stem cells (DPSCs), embryonic stem (ES) cells (ESCs), and multilineage-differentiating stress-enduring cells (Muse cells) (Chen et al., 2001; Yang et al., 2016; Niizuma et al., 2018; Rikhtegar et al., 2019). The potential mechanisms underlying the protective role of stem cells have been extensively studied, such as the promotion of angiogenesis and endogenous neurogenesis, immunomodulatory functions, differentiation into cells that facilitate repair or replacement of a damaged tissue, secretion of cytokines helping to restore neural injury, and cell migration (Gutiérrez-Fernández et al., 2013; Shichinohe et al., 2015; Tan et al., 2018; Rikhtegar et al., 2019; Kikuchi-Taura et al., 2020). This review summarizes and discusses the pathophysiological changes after IS, which are also the treatment targets of stem cell therapy, the current types of cell therapy, the stem cell used, and the neuroprotective mechanisms. Clinical studies on stem cell stroke therapy are discussed along with the choice of stem cell types, time of transplantation, route of administration, and cell dose, as these are regarded as controversial and worrying aspects by clinicians and neuroscientists.

## Potential Therapeutic Targets of Stem Cell Therapy in Ischemic Stroke

Ischemic stroke accounts for 71% of strokes and is the treatment target in most stroke trials (Feigin et al., 2018). In the past decades, the pathophysiological changes after stroke have been extensively investigated. Understanding the changes in the microenvironment and cell activities that occur after IS can help to focus on the potential effective role of stem cell therapy, and the pathophysiological changes may also be potential stem cell therapy targets.

### Blood–Brain Barrier Disruption

The BBB plays a vital role in the protection of the central nervous system (CNS) (Daneman and Prat, 2015). The basic structure of the BBB consists of endothelial cells (ECs), astrocytes, and pericytes, with the ECs fused together by intercellular junctions such as tight junctions (TJs) and adherent junctions (AJs). After the onset of stroke, the BBB can be disrupted, followed by the extravasation of blood components into the brain, thus compromising the normal neuronal function. BBB dysfunction is characterized by the structural disruption of the intercellular junctions and increased vascular permeability, which allows the components of the peripheral blood system to cross into the parenchyma more easily. After IS, BBB permeability is increased and cell adhesion molecules are upregulated in the activated endothelium, and peripheral immune cells can access the parenchyma. The infiltrating leukocytes can contribute to BBB dysfunction and injury progression (Shi et al., 2019), and neutrophils are another important peripheral immune cell type that can cross the damaged BBB. In addition to the release of chemokines (Nathan, 2006), activated neutrophils can increase the formation of neutrophil extracellular traps (NETs), which contain double-stranded DNA, histone, and granule proteins including neutrophil elastase, cathepsin G, and myeloperoxidase (MPO) (Urban et al., 2009). The latest research suggests that reduced neovascularization and increased BBB damage can be observed under the increased formation of NETs induced by the overexpression of peptidylarginine deiminase 4, while the inhibition of this process by DNase 1, genetic ablation, or pharmacologic administration improves BBB function (Kang et al., 2020).

### Neuroinflammation and Immune Response

Neuroinflammation after stroke is a sterile inflammation defined as an inflammatory response by cells of the innate or adaptive immune systems within the CNS. In IS, neuronal and non-neuronal cell death can produce damage-associated molecular patterns (DAMPs) because of oxygen and glucose deprivation (OGD). During ischemia, DAMPs can activate astrocytes and microglia, increasing production of pro-inflammatory cytokines and chemokines. Peripheral blood then invades the infarcted area, leading to the exacerbation of tissue damage (Dabrowska et al., 2019a). After DAMPs are released into the brain parenchyma, microglia cells are the first line of defense. They secrete pro-inflammatory cytokines, such as tumor necrosis factor-α (TNF-α), NF-κB, interleukin (IL)-1β, IL-12, IL-23, and nitrogen monoxide (NO), which induce tissue damage (Benakis et al., 2014; Xiong et al., 2016). Recently the inflammasome, which is a multi-molecular protein complex and one of the intracellular typical pattern recognition receptors (PRRs) located in neurons, microglia, astrocytes after IS, has attracted a lot of attention. The members of the nucleotide-binding domain (NOD)-like receptor (NLR) family including the NODs, the NLRPs, and the IPAF subfamilies exhibit inflammasome activity (Mohamed et al., 2015). The NLRP3 inflammasome can cleave pro-IL-1 and pro-IL-18, which become mature IL-1 and IL-18 (Alishahi et al., 2019). Neutrophils are among the first cells to arrive into the lesion within the first hour after IS (Garcia et al., 1994) and leukocytes invading the CNS release pro-inflammatory factors in the ischemic region. Neutrophils increase the recruitment of leukocytes by degranulation of their content rich in cytokines/chemokines, free radicals, proteolytic enzymes, and activated-complement system, which in turn exacerbates neuroinflammation (Mayadas et al., 2014). Recently, the detrimental role of neutrophils in IS has been demonstrated. The release of pro-inflammatory cytokines/chemokines, proteases, and oxygen radicals (Neumann et al., 2015), combined with the antagonistic effect of some chemokines such as C-X-C motif chemokine receptor 2 (CXCR-2) and (C-X-C motif) ligand 1 (CXCL-1), can prevent neutrophil recruitment into the infarcted area and reduce the experimental infarcted volume (Gelderblom et al., 2012; Herz et al., 2015).

### Excitotoxicity

Excitotoxicity was first described in 1986 to describe the ability of glutamate and structurally related excitatory amino acids to destroy neurons (Olney, 1969). Glutamate is the main excitatory neurotransmitter in the CNS. The OGD resulting from the interruption of blood flow in IS disrupts ATP supply, which leads to perturbation of the transmembrane gradient, impairment of the neuronal signaling, and neurotransmitter release as a result of anoxic depolarization (Obrenovitch et al., 1993). Depolarization leads to a transient neurotransmitter release, and the reuptake of excitatory neurotransmitters from the synaptic cleft is an ATP-dependent process. Therefore, ischemia leads to an increase in extracellular neurotransmitter concentrations. *N*-Methyl-D-aspartate (NMDA) is a glutamate receptor that plays a key role in excitotoxicity, and in a normal physiological state, its function is controlled by extracellular magnesium and the resting membrane potential. However, after IS, neuronal depolarization removes magnesium, and the NMDA receptor is activated, leading to calcium influx. The primary influx then induces the secondary release of large amounts of calcium from the intracellular environment leading to cell death (Choi, 1992; Lai et al., 2014; Chamorro et al., 2016). To deal with the high concentration of intracellular calcium, mitochondria sequester the majority of the intracellular calcium after glutamate excitotoxicity (Wang and Thayer, 1996). The increase in the glutamate concentration can also activate the α-amino-3-hydroxy-5-methyl-4-isoxazolepropionic acid (AMPA) receptors, which in normal conditions are not calcium permeable (Peng et al., 2006), but after IS, permeability increases up to 18-fold, contributing to calcium-dependent cell death (Liu et al., 2006).

### Oxidative Stress

After the onset of IS, the balance between pro-oxidants [such as reactive oxygen species (ROS)] and antioxidants is compromised, leading to oxidative stress and damage of the cell structures, including lipids, proteins, and DNA, finally leading to cell death (Zhao et al., 2016). ROS greatly contributes to lipid peroxidation after IS by increasing the production of conjugated dienic hydroperoxides (Adibhatla and Hatcher, 2010) and can decompose the ω-6 polyunsaturated fatty acids into 4-hydroxynonenal (4-HNE), a traditional marker of oxidative stress (Niki, 2009). 4-HNE can enhance apoptosis/autophagy and increase the cerebral infarct area (Lee et al., 2012). Phospholipase A2 (PLA2) is another contributor to lipid peroxidation that can lead to the hydrolysis of membrane phospholipids and the release of free fatty acids. At a high ROS concentration, lipid peroxides are formed and degraded into active aldehydes, which can bind to proteins or nucleic acids, causing neuronal damage (Muralikrishna Adibhatla and Hatcher, 2006). The products of lipid peroxidation such as malondialdehyde (MDA), 4-HNE, and acrolein participate in oxidative stress injury on proteins (Del Rio et al., 2005; Moghe et al., 2015). Oxidative stress can also cause cell death by damaging the DNA (Li et al., 2011). This damage activates the DNA repair signal, which includes the activation of poly(ADP-ribose) polymerase-1 (PARP-1). To repair the DNA strand, β-nicotinamide adenine dinucleotide (NAD^+^) is consumed by PARP-1, which can result in impairment of NAD^+^-dependent processes, including mitochondrial respiration, leading to ATP starvation and, eventually, neuronal death (Southan and Szabó, 2003).

## Cell Therapy

Because IS involves complex pathophysiological processes, one targeted therapeutic strategy is far from enough. The use of cell therapy, including stem cells, gene-modified stem cells, and polarized cell therapy, has been widely discussed in preclinical or clinically studies. After the onset of IS, CNS cells cannot completely replace the lost or compromised ones because of their limited regeneration and differentiation ability. Stem cells have emerged as a novel and promising option due to their capacity for self-renewal, homing, and multilineage differentiation (Birbrair, 2017; Huang et al., 2018). A variety of stem cell types derived from neural and non-neural tissues have been widely studied as potential donors for IS treatment.

Neurotrophic factor secretion is an important mechanism underlying the function of stem cell therapy in IS. Growth factors such as brain-derived neurotrophic factor (BDNF), vascular endothelial growth factor (VEGF), and erythropoietin (EPO) are involved in the restoration of the damaged tissue to prevent neuronal death (Schäbitz et al., 2000), angiogenesis, anti-inflammation, or brain repair after IS. Therefore, gene-modified stem cells, which can overexpress different growth factors or cytokines through gene transfection, will possess a unique superiority in terms of injury repair compared with unmodified stem cells (Geiseler and Morland, 2018).

The phenotypes of several CNS cells, such as microglia, macrophages, astrocytes, and neutrophils, are in a dynamic flux and can thus exhibit two different phenotypes: the classic pro-inflammatory type and alternative protective type. After the onset of IS, the pro-inflammatory phenotype cells increase inflammatory mediators inducing brain tissue injury (Block et al., 2007; Kanazawa et al., 2017b; Liddelow and Barres, 2017), while the anti-inflammatory phenotype cells can secrete neurotrophic factors and protective cytokines (Imai et al., 2007; Crain et al., 2013), which have a neuroprotective function (Schäbitz et al., 2000; Hu et al., 2012; Dudvarski Stankovic et al., 2016; Geiseler and Morland, 2018). When considering classical or gene-modified stem cells, ethical issues and tumorigenicity must be taken into account, as highlighted in previous studies (Murry and Keller, 2008; Ratajczak et al., 2019). Targeting microglia or other polarized cells after IS may be an optional cell therapy strategy. Because of the lack of relevant research, the specific protective function should be a focus of future studies (Hatakeyama et al., 2020b).

## Different Stem Cell Types in Cell Therapy

### Mesenchymal Stem Cells

Mesenchymal stem cells have the capacity for self-renewal and potential multidirectional differentiation into different cell types and are available from almost any tissue type. The bone marrow-derived MSCs (BM-MSCs) are the most widely studied and are capable of differentiating into neurons or glial cells and, thus, are able to replace damaged cells in brain tissues. The ability to differentiate into ECs and release trophic factors such as VEGF, BDNF, glial cell-derived neurotrophic factor (GDNF), and transforming growth factor (TGF), contribute to angiogenesis and neuroprotection (Wakabayashi et al., 2010; Wagenaar et al., 2018). Although MSCs have a high ability to differentiate into neuronal lineage cells and migrate into ischemia lesions, only a small proportion of stem cells enter the infarct core area (Modo et al., 2004). The paracrine action or “bystander effect,” which relies on the secretion of trophic factors, is the main contributor to the therapeutic effect rather than direct cellular replacement. The trophic factors released from MSCs promote angiogenesis, induce the proliferation and recruitment of endogenous stem cells from the subventricular zone (SVZ) to the infarcted area, and modulate neuroinflammation. MSCs also promote the production of regulatory T cells (Wang et al., 2014), which increase the expression of IL-10, attenuate astrocytes and microglial reactivity, exert an anti-apoptotic effect, and reduce levels of the inflammatory cytokines IL-1β and IL-6 (Zhu et al., 2014; Sotomayor-Sobrino et al., 2019).

### Multilineage-Differentiating Stress-Enduring Cells

Multilineage-differentiating stress-enduring cells are characterized by self-renewal and multipotency and can differentiate into all three germ layers (Wakao et al., 2011; Heneidi et al., 2013; Dezawa, 2016). MUSE cells are found in the bone marrow, adipose, dermis, and connective tissues (Heneidi et al., 2013; Gimeno et al., 2017) and can be purchased or collected from human tissues. After the first report on MUSE cells in 2010 (Kuroda et al., 2010), several other studies have investigated their therapeutic potential in diseases such as acute myocardial infarction (MI), liver disease, and chronic kidney disease (CKD) (Katagiri et al., 2016; Yamada et al., 2018). In normal conditions, MUSE cells are inactive. After IS, the ischemia insult stimulates MUSE cell mobilization from the bone marrow into the peripheral blood flow (Hori et al., 2016). MUSE cells effectively migrate into the infarcted area where they differentiate into neural cells to restore the damaged brain tissues. Their efficient migration has also been observed in other diseases including MI, CKD, and liver disease (Uchida N. et al., 2017; Yamada et al., 2018). After reaching the ischemic area, MUSE cells can replace the damaged cells by differentiating into neuronal-lineage cells (Uchida et al., 2016; Abe et al., 2020). The administration of diphtheria toxin (DT) in a rat IS model inhibits recovery, suggesting that the neural circuit reconstruction is a key mechanism underlying the therapeutic effects (Uchida H. et al., 2017; Abe et al., 2020). MUSE cells can also modulate neuroinflammation and immune response by downregulating the secretion of pro-inflammatory cytokines including TNF-α (Gimeno et al., 2017). While tumor formation and other adverse effects have not been reported, some issues remain to be solved, including the appropriate timing, dose, and safety, before their use in clinical practice can progress.

### Neural Stem Cells

Neural stem cells are self-renewing cells and possess the multi-potential to differentiate into neurons, astrocytes, and oligodendrocytes (Boese et al., 2018; Obernier and Alvarez-Buylla, 2019; Ottoboni et al., 2020). Exogenous NSCs can be derived from ESCs, iPSCs, fetal tissue, and adult nervous systems. Their abilities to produce primary CNS cells make NSCs promising candidates for the replacement of the damaged or lost cells in the brain tissues after IS. Under normal conditions, neurogenesis persists throughout the whole life in rodents and humans (Boldrini et al., 2018), and endogenous NSCs, found mainly in the dentate gyrus of the hippocampus, the SVZ, and the olfactory bulb in mammals, remain inactive (Obernier and Alvarez-Buylla, 2019). After IS, cytokines or chemokines such as stromal cell-derived factor-1 (SDF-1), VEGF, and monocyte chemoattractant protein (MCP)-1 are released, increasing the proliferation and migration of NSCs from the SVZ into the ischemic area (Mine et al., 2013; Leal et al., 2017; Geiseler and Morland, 2018). Thanks to their strong ability to differentiate into neuronal cells, transplanted NSCs have been shown to be effective in promoting the neurological function recovery in preclinical studies. NSCs can migrate into the ischemic brain area and differentiate into mature neurons to reconstruct the neural circuit (Shetty and Hattiangady, 2016). Exogenous NSCs are also capable of stimulating neurogenesis in the SVZ. NSCs release neurotrophic factors such as BDNF, VEGF, and nerve growth factor (NGF), which can prevent neural apoptosis and improve functional recovery by directly or indirectly increasing the angiogenesis, endogenous neurogenesis, and plasticity of neuronal cells (Shetty and Hattiangady, 2016; Ottoboni et al., 2020). The activation of microglia and infiltration of neutrophils also decrease after NSC transplantation, thus relieving neuroinflammation (Lee et al., 2008).

### Induced Pluripotent Stem Cells

Induced pluripotent stem cells are somatic-derived stem cells characterized by an increased translational potential. iPSCs were first established in 2006 by introducing transcription factors into mouse fibroblasts during a pluripotent state (Takahashi and Yamanaka, 2006). This somatic cell-derived feature also gives iPSCs the following advantages over other stem cells: (i) they can be obtained from different sources, and isolation does not require invasive measures; (ii) iPSC culture provides an adequate cell number for transplantation in a shorter time; and (iii) autologous cells show almost no immunogenicity after transplantation (Ojeh et al., 2015; Vitrac and Cloëz-Tayarani, 2018). Furthermore, iPSCs have similar advantages to ESCs in that they are pluripotent, can undergo self-renewal, and can differentiate into cells from three germ layers (Takahashi and Yamanaka, 2006; Takahashi et al., 2007). While their differentiation into neurons, ECs, or astrocytes may improve the overall effect of the treatment, there is also a risk of tumorigenicity (Suda et al., 2020).

### Dental Pulp Stem Cells

Dental pulp stem cells are ectoderm-derived stem cells residing within the dental pulp (Nosrat et al., 2004), which can differentiate into multiple cell types including neural cells, muscle, cartilage, and adipocytes (Dailey et al., 2013). They have similar properties to both NSCs and MSCs in terms of surface markers and biological features (Gronthos et al., 2000; Martens et al., 2013). The expression of nestin, MAP2, the astrocytic marker GFAP, and NeuN by DPSCs has also been reported (Gronthos et al., 2002; Sakai et al., 2012; Foudah et al., 2014), suggesting that they have the potential ability to differentiate into neuron-like cells. In an animal model of spinal cord injury (SCI), transplanted DPSCs improved functional recovery by three mechanisms: (1) decreased CNS cell apoptosis; (2) their paracrine effect minimized the influence of multiple axon growth inhibitors such as chondroitin sulfate proteoglycan and myelin-associated glycoprotein; and (3) they differentiated into mature neural cells and replaced the lost brain tissues (Sakai et al., 2012). DPSCs can also differentiate into dopaminergic neurons and reconstruct immature neural networks (Chun et al., 2016). While they have properties similar to those of MSCs and NSCs, they confer more advantages, their proliferation is significantly faster than that of MSCs (Ponnaiyan and Jegadeesan, 2014), and they can also more effectively reduce the infarcted area and improve motor function compared with MSCs (Song et al., 2017). Moreover, DPSCs secrete specific neurotrophic factors such as GDNF, NGF, and BDNF; and the conditioned medium containing these factors is also a promising novel treatment for IS (Kichenbrand et al., 2019). Finally, DPSCs protect neural cells from oxidative stress by decreasing OGD-induced ROS production (Song et al., 2015) and contributing to immune system modulation by inhibiting T cell activation and increasing CD3^+^ T cell apoptosis (Pierdomenico et al., 2005; Demircan et al., 2011).

### Hematopoietic Stem Cells (CD34 Positive)

Hematopoietic stem cells are a rare cell population obtained from the bone marrow, mobilized peripheral blood (MPB), and umbilical cord blood (UCB) (Behzad-Behbahani et al., 2005; Sovalat et al., 2011). HSCs are able to self-renew and can differentiate into a full spectrum of blood cells (Derakhshani et al., 2019). CD34^+^ HSCs were found to invade the peripheral blood after acute stroke (Hennemann et al., 2008). In published studies, HSCs are almost all CD34 positive (Uemura et al., 2012) and under normal conditions maintain a quiescent state via the coupling of CXCL12 with CXCR4. After IS, the increase in sympathetic tone and upregulation of granulocyte colony-stimulating factor (G-CSF) promote the mobilization of CD34^+^ HSCs, which are recruited to the injured lesion where they can exert a protective effect (Borlongan et al., 2011). CD34^+^ HSCs from bone marrow and UCB enhance the neuroprotective ability through angiogenesis, neurogenesis, and the regulation of inflammation (Hennemann et al., 2008). HSCs are also feasible, safe, and effective in the treatment of hematologic, cardiovascular, bone, and genetic defect diseases (Borlongan et al., 2011). This may be related to their inability to undergo neuronal differentiation and are thus unable to complete the complex restoration process necessary to repair the damage after IS. However, HSCs can improve neuronal structures through fusion with their residential components and adoption of their phenotype (Terada et al., 2002).

### Embryonic Stem Cells

Embryonic stem cells are isolated from early embryonic or primitive gonads, are immortalized *in vitro*, and possess self-renewal and multilineage differentiation abilities (Murry and Keller, 2008). ESCs can be induced to differentiate into almost all somatic cell types when cultured with specific cytokines (Ratajczak et al., 2019). After being considered cells of unlimited potential for regenerative medicine, a variety of problems emerged, causing widespread concern around the world. The first is the ethical issue, which is constantly debated, but other concerns exist, such as tumorigenicity, immune rejection, induction of epigenetic changes, and genetic alterations, which have also been discussed (Murry and Keller, 2008; Ratajczak et al., 2019). It is worth pointing out that the tumorigenicity of ESCs can be reduced by modulating the anti-apoptotic Bcl-2 gene to regulate epigenetic stability (Allegrucci et al., 2007).

Very little research on ESC transplantation in stroke has been done. Striatal transplantation of mouse ESCs in a rat IS model was shown to alleviate neurological dysfunction by improving dopaminergic function (Yanagisawa et al., 2006). Other animal experiments have revealed that ESCs exert a protective effect on cerebral ischemia after generating secondary cells such as neural precursors, neural lineage cells, and NSCs after differential induction *in vitro*. These inductions can be caused by neurotrophic factors such as BDNF, small molecule inhibitors, co-culture with other cells like bone marrow stromal cells (BMSCs), or other means (Yang et al., 2009; Drury-Stewart et al., 2013; Rosenblum et al., 2015). Although clinical trials in the United States have used ESCs to treat SCI (Ilic et al., 2015), their use is not widespread due to theoretical and technical difficulties, as well as public opinion.

While different stem cell types have similarities, or overlaps in their protective functions, they each have particular strengths and limitations. MSCs are one of the most studied stem cells; they have a rich research knowledge base, have strong differentiation ability, and are easy to collect but have limited ability to enter the infarct core area. Other stem cells such as MUSE cells and NSCs can efficiently enter the infarct area and reconstruct the neuronal structure. In current research examining the protective function of a specific stem cell, researchers pay greater attention to the role of the “bystander effect,” exosomes, and paracrine effect and, thus, to some extent, ignore the basic characteristics of stem cells such as the ability of differentiating into mature cells, which can directly replace the damaged brain tissue. In MUSE cells, iPSCs, and ESCs, the focus has been less on bystander effects and more on their strong ability to differentiate and replace damaged cells and rebuild the neuronal circuit; however, ethical issues and tumorigenicity must be taken into consideration in further ESCs or iPSC research. The role of stem cells is not limited to one cell or mechanism, and there are more stem cell types and mechanisms for us to discover and explore. Further study on induced differentiation, gene-edited stem cells, or finding new stem cell types will greatly advance our current knowledge base.

## Protective Mechanisms Regulating Stem Cell Therapy After IS

Stem cell transplantation can improve the IS outcome from different aspects, exerting a variety of protective effects thanks to the pleiotropic mechanisms underlying stem cell therapy. Over the last decades, several stem cell types have been widely studied and even been used in clinical practice. Mechanisms to regulate their therapeutic effects include modulation of neuroinflammation and the immune response, angiogenesis, neurogenesis, secretion of protective factors, and cell migration.

### Stem Cell Migration

The first step in restoring damaged cells following stroke is the migration of stem cells into the damaged brain regions (De Feo et al., 2012); however, it is not yet clear how the stem cells enter the infarct core or boundary area after IS; therefore, modulating their migration remains a challenge in translating stem cell therapy from the laboratory to clinical practice (Sullivan et al., 2015). Understanding this mechanism should be a critical part of future studies on the protective effects of stem cell therapy.

Although endogenous NSCs located in the SVC remain inactive in normal conditions (Obernier and Alvarez-Buylla, 2019), the onset of IS is associated with its activation and recruitment to the adjacent lesioned areas (Nakayama et al., 2010), and the ischemic insult also leads to increased migration, proliferation, and differentiation. During the ischemia condition, the hypoxia-inducible factor-1α (HIF-1) represents an important transcriptional factor induced by hypoxia itself, which can promote neuroprotection during a particular time window (Yamashita and Abe, 2016). HIF-1 can upregulate EPO release (Li et al., 2020a), which contributes to stem cell migration after IS, while the knockdown of EPO receptors significantly decreases post-stroke neurogenesis by impairing stem cell migration (Tsai et al., 2006). The upregulation of EPO increases BDNF expression, which is neuroprotective in IS. A recent study revealed that although BDNF administration has no effect on migration speed of stem cells, BDNF treatment of NSCs can result in significant chemotactic and directional migration in CXXL12 gradients compared with untreated NSCs (Xu and Heilshorn, 2013). Due to the role of BDNF in stem cell migration, recent research has shown that a certain level of BDNF can serve as a minimum predictive value for the recovery of motor function after IS (Luo et al., 2019). The mesencephalic astrocyte-derived neurotrophic factor (MANF) also plays a crucial role in regulating neural progenitor cell (NPC) migration and protects endogenous NSCs against OGD-induced injury (Tseng et al., 2018).

As discussed above, the BBB is a selective barrier composed of ECs, pericytes, and astrocytes; and the TJs and AJs between ECs help the BBB to separate the CNS from the peripheral blood flow. The entrance of transplanted stem cells from the peripheral blood circulation to the brain parenchyma through the BBB is therefore necessary for stem cells to exert an effective therapeutic effect. The expression of E- and P-selectin in the cerebral microvasculature increases after IS (Huang et al., 2000). A recent study observed increased adhesion of BMSCs in cerebral venules after the IS compared with sham mice, and the immunoneutralization of either E- or P-selectin blocks the recruitment of adherent BMSCs (Yilmaz et al., 2011). These results suggest that ECs exhibit a pro-adhesive phenotype that increases selectin-dependent rolling and adhesion after IS. VCAM-1 is also an adhesion molecule upregulated on EC membrane after IS. Transplanted NSCs can adhere to the VCAM-1 in a static adhesion assay, and CD49 may serve as the critical ligand for VCAM-1-mediated NSC adhesion (Guzman et al., 2008). Laminin signaling can increase the migration of neuroblasts along vascular scaffolds to injured areas via β1 integrin (Fujioka et al., 2017). Selectin-mediated rolling and integrin-associated adhesion play important roles in the transendothelial processes of transplanted stem cells, although they can also increase the recruitment and infiltration of neutrophils after the onset of IS, which can aggravate brain damage (Ao et al., 2018); however, its detailed mechanisms need to be further elucidated. After stem cell adhesion to the ECs, CXCL-11 is released, which can bind CXCR-3 and increase the permeability of BBB by opening the TJs under the activation of ERK1/2 signaling pathway (Feng et al., 2014). Stem cell therapy can also reduce BBB disruption by preventing the upregulation of MMP-9 and downregulating ICAM-1 in ECs (Cheng et al., 2018). The apparent opposite effect of stem cells on the BBB depends on the different stages of stem cells, an aspect that requires further exploration.

**FIGURE 1:**
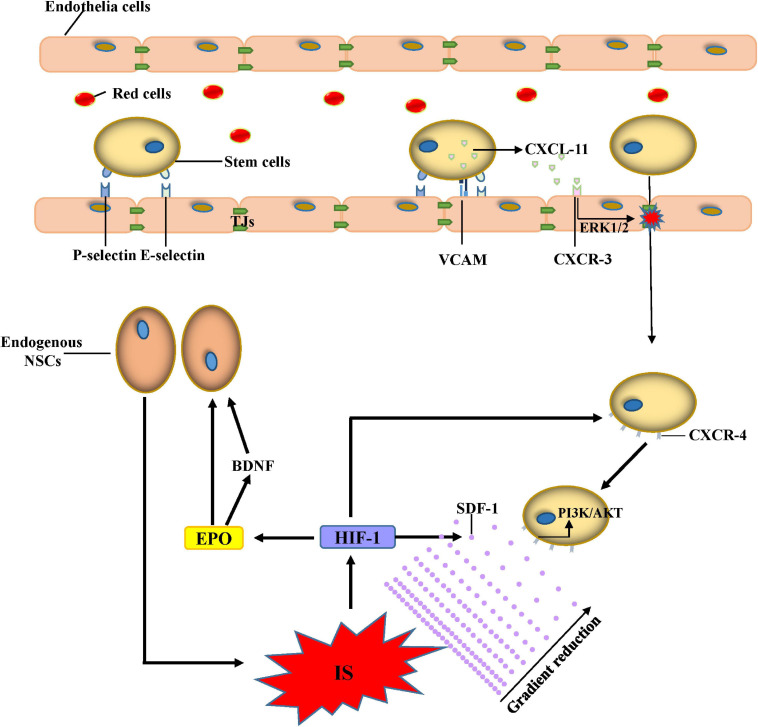
Mechanisms of stem cell migration. Adhesion between stem cells and endothelial cells increases after IS due to the increased expression of E-selectin, P-selectin, and VACM. CXCL-11 released from stem cells can bind CXCR-3 and increase the permeability of the BBB by opening the TJs under the activation of the ERK1/2 signaling pathway. Administered stem cells can migrate to the ischemic lesion by the gradient of SDF-1 through the activation of the PI3K/AKT signaling pathway. The proliferation and migration of endogenous NSCs increase due to BDNF and EPO after IS. Abbreviations: IS, ischemic stroke; VCAM, vascular cell adhesion molecule; CXCL, C-X-C motif ligand; CXCR, C-X-C motif chemokine receptor; EPO, erythropoietin; HIF, hypoxia-inducible factor; SDF, stromal cell-derived factor; PI3K, phosphoinositide-3-kinase; NSCs, neural stem cells; TJs, tight junctions.

After IS, HIF-1 stimulates the transcription of SDF-1/CXCL12 (Ceradini and Gurtner, 2005); and the levels of CXC chemokine receptor 4 (CXCR4), an SDF-1 receptor, increase on the membrane of stem cells (Delcroix et al., 2010; Moll and Ransohoff, 2010). SDF-1 binding to CXCR4 activates several signaling pathways such as the phosphatidylinositide-3-kinase (PI3K/Akt), ERK1/2, c-Jun N-terminal kinase (JNK), and p38 MAPK pathways, which enhance stem cell migration. The extent to which pathways are activated is influenced by several factors such as the differentiation stage (Chen Y. et al., 2014) and the concentration of SDF-1 (Yu et al., 2016). Despite the interaction between SDF-1 and CXCR4, a recent study found that osteopontin (OPN) is a phosphoglycoprotein constitutively expressed in the brain, which plays an important role in tissue homeostasis. OPN expression upregulates and stimulates the migration of NSCs via CXCR4, resulting in an effective therapeutic effect after IS (Rabenstein et al., 2015). During ischemia, C-C motif chemokine ligand 2 (CCL2), one of the most highly expressed chemokines, contributes to neurological repair; and the interaction between CCL2 and CCR2 (CCL2 receptor) enhances stem cell migration to the infarcted area, promoting subsequent brain repair (Huang et al., 2018; Lee et al., 2020). TLRs are important mediators that cause neuroinflammation but are also involved in brain repair after IS. The ischemic insult induces TLR4 activation, which is involved in the early expression of SDF-1 and faster recovery of BDNF expression, which promotes migration of endogenous neuroblasts (Palma-Tortosa et al., 2019). Neuroinflammation is a crucial aspect of IS pathophysiology; and inflammatory chemotactic agents and cytokines such as MCP-1, macrophage inflammatory protein-1alpha (MIP-1alpha), and IL-8 expressed in the injured brain can increase stem cell migration into the infarcted areas (Wang et al., 2002; Figure 1).

### Modulation of Neuroinflammation and Immune Response

After the onset of IS, the DAMPs released from the damaged CNS cells bind receptors such as TLRs or nuclear receptors to activate the innate immune system (Benakis et al., 2014), and increase expression of pro-inflammatory cytokines. As previously summarized, the cascade is mediated by the activation of innate immune cells or leukocytes from the peripheral blood infiltrating through the damaged BBB. Transplanted stem cells can ameliorate the inflammatory process and exert a protective function.

To deal with the cascade, transplanted stem cells can decrease the expression of pro-inflammatory cytokines. MSC added to conditioned media can reduce the secretion of IL-6 and TNF-α by microglia (Dabrowska et al., 2019a; Tobin et al., 2020). The reduction of IL-1β, IL-6, and TNF-α can also be observed in MSC-treated mice following ischemia (Cheng et al., 2018). Other stem cell types, including NSCs, DPSCs, and iPSCs, can also protect the CNS from damage by pro-inflammatory cytokines (Eckert et al., 2015; Nito et al., 2018). Stem cells can also secrete anti-inflammatory cytokines including IL-4, IL-10, and TGF-β1 (Zhang et al., 2016).

In addition to the important role of ameliorating neuroinflammation, different cell types involved in neuroinflammation or the immune response such as macrophages, microglia, astrocytes, and even some antigen presenting cells can also be a stem cell target. DPSCs, for example, possess a potent immunoregulatory function by increasing CD3^+^ T cell apoptosis and the expression of anti-inflammatory cytokines and by decreasing the expression of pro-inflammatory cytokines (Demircan et al., 2011). BMSCs can also decrease infiltration of gamma delta T (γδT) cells and increase infiltration of regulatory T cells (Tregs) after IS (Wang et al., 2014). Macrophages co-cultured with MSCs exhibit a phenotype with low secretion of IL-6 and TNF-α and high expression of IL-10, which is similar to an anti-inflammatory phenotype, indicating that stem cells can modulate the immunophenotype and functional characteristics of macrophages (Kim and Hematti, 2009). MSCs can reduce proliferation of NK cells, cytokine secretion, and the expression of receptors by similar regulatory mechanisms (Valencia et al., 2016). After the onset of IS, the activation of residual cells in the CNS plays an important role in neuroinflammation. NSC transplantation decreases the number of infiltrating microglia, macrophages, inducible nitric oxide synthase (iNOS), and cyclooxygenase (COX)-2 expressing cells (Watanabe et al., 2016) and also reduces cytokine levels (Lee et al., 2008). Furthermore, iPSC transplantation in an SCI model not only reduces the activated macrophages or microglia but also inhibits GFAP-positive cells and glial scar formation. The increasing expression of IL-10 after stem cell transplantation partly accounts for the attenuation of astrocyte reactivity.

**FIGURE 2:**
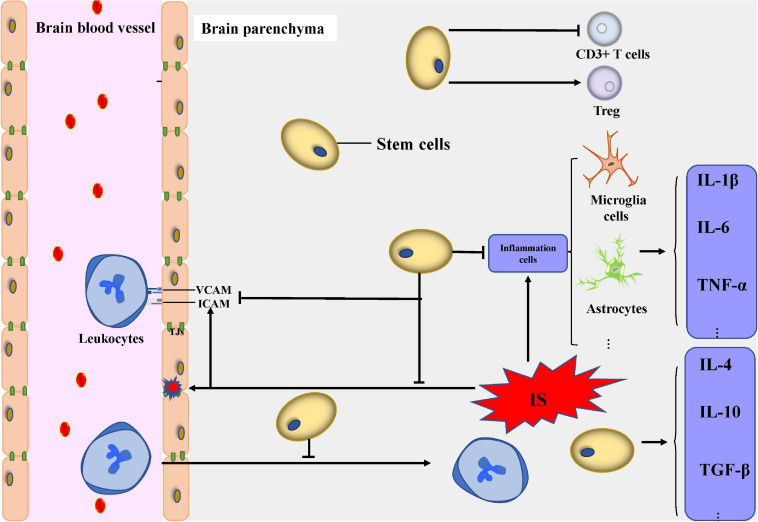
Modulation of neuroinflammation and immune response. Transplanted stem cells can decrease the expression of pro-inflammatory cytokines including IL-1β, IL-6, and TNF-α and secrete anti-inflammatory cytokines including IL-4, IL-10, and transforming growth factor-β1. Transplanted stem cells can modulate the activity of inflammatory cells including microglial cells, astrocytes, and T cells. The infiltration and pro-inflammation effect of leukocytes from peripheral blood decreased under the administration of stem cells. Abbreviations: IS, ischemic stroke; VCAM, vascular cell adhesion molecule; ICAM, intercellular adhesion molecule; IL, interleukin; TNF, tumor necrosis factor; TGF, transforming growth factor; Treg, regulatory T cells; TJs, tight junctions.

In the acute phase of IS, leukocytes invade the brain parenchyma due to the increased BBB permeability and the upregulation of ICAM and VCAM. The results of histologic quantifications in the peri-stroke areas revealed that NSC transplantation decreases MPO^+^ neutrophil infiltration (Lee et al., 2008). Another study revealed that NSC transplantation downregulates ICAM and VCAM, which can, to some extent, repair the BBB (Huang et al., 2014). However, some stem cells cannot directly improve inflammation and immune response by targeting inflammatory factors and immune cells, or maybe their exact mechanism has not yet been discovered. In the chronic phase of IS, some stem cells can indirectly improve neovascularization, neurogenesis, and restoration of BBB, thus exerting anti-inflammatory effects. The recent increase in interest in human pluripotent stem cells (hPSC) is due to the fact that they have the potential to differentiate into cells with BBB markers and characteristics (Lippmann et al., 2020); thus, their protective effect could be partly due to their ability to restore the BBB. In light of all these discoveries, stem cell therapy represents a potential treatment to protect the CNS through neuroinflammation activity (De Feo et al., 2012; Figure 2).

### Secretion of Protective Factors

Some recent studies have found a lower density of transplanted stem cells in the stroke core compared with the penumbra or surrounding area, suggesting that functional improvements are more likely mediated by the release of trophic factors rather than cell replacement and differentiation, a phenomenon known as the “bystander effect.”

The bystander effect is common in the protective mechanisms of stem cell used in the IS therapy. The use of GRID, a contrast agent that allows monitoring of NSCs, revealed that these stem cells migrate to the area around the ischemic infarct instead of the core area in rats (Modo et al., 2004). The change of function and structure of the neuron network was non-invasively monitored using MRI for 3 months after stem cell implantation in rats. It is that revealed the functional network sharply decreased for the entire 3 months, while it was previously stabilized in mice with NSC transplantation, indicating the important paracrine role of stem cell treatment in IS therapy (Green et al., 2018). DPSCs can move into the boundary area of the ischemic lesion and mostly differentiate into glial cells instead of neurons (Song et al., 2017); however, trophic factors such as cytokines, chemokines, and exosomes released by the stem cells can interact with different cells in the CNS to improve the outcome after IS. These factors are released into the microenvironment via direct permeation and extracellular vesicles (EVs) such as exosomes or microvesicles and exert a paracrine effect. In recent years, many studies have shown that microglia, astrocyte, and macrophage activation can be effectively attenuated by neurotrophic factor secretion (Valencia et al., 2016; Watanabe et al., 2016) after stem cell transplantation. The secretion of high levels of protective cytokines and neurotrophic and angiogenic factors is involved in the paracrine effect after IS (Stonesifer et al., 2017; Geiseler and Morland, 2018), and this effect on stem cells and its role in IS have been recently investigated. After injection with a conditioned medium rich in MSCs, paracrine factors into the lateral ventricle of the middle cerebral occluded artery of rats, the infarct volume and cerebral edema were significantly reduced, and the neurological deficits were improved (Asgari Taei et al., 2020). In rats receiving transplanted stem cells, human insulin-like growth factor 1 (IGF-1) was present in the peri-infarcted area at 3 days, and they had a higher level of neurotrophic factors such as VEGF, epidermal growth factor (EGF), and basic fibroblast growth factor (bFGF) than the control group (Wakabayashi et al., 2010).

Extracellular vesicles have recently received a great deal of recent attention. They are membrane vesicles containing proteins, nucleic acids, and bioactive lipids that can be secreted by different types of cells (Raposo and Stoorvogel, 2013). After being released from the original cells, EVs enter the neuronal microenvironment; and the content of the original cells, such as membrane and cytosolic lipid, protein, and RNA, is transferred. EVs serve as a mediator, participating in intercellular communication and finally leading to epigenetic changes and functional modifications of neighboring or distant cells. The expression of VEGF and HIF-1α is upregulated in the core area and ischemic border zone (IBZ) after MSC transplantation, which can increase angiogenesis. When the human microglia cell line (HMO6) and MSCs are co-cultured *in vitro*, VEGF mRNA increases in both lines, indicating that MSCs increase angiogenesis by a paracrine effect (Sheikh et al., 2019). EVs may be the main intercellular mediator, and those secreted by MSCs can enhance angiogenesis by activating the signal transducer and activator of transcription 3 (STAT3), the suppression of which can abolish the paracrine effect (Xia et al., 2020). EVs from stem cells are rich in various microRNAs (miRNAs), which can target the 3’-untranslated region (UTR) to upregulate VEGF, bFGF, and HIF and activate the PI3K/AKT, Ras/Raf/MEK/ERK/MAPK, and Notch signaling pathways, all of which are associated with angiogenesis (Zhu et al., 2015; Guo et al., 2018; Potz et al., 2018; Icli et al., 2019). Due to the lower migration of the transplanted adipose-derived stem cells (ADSCs) into the ischemic core area, a study aiming to discuss the potential paracrine effect of stem cell therapy after IS found that EVs mediate the transfer of miRNA-126, which significantly increases the expression of von Willebrand factor (an EC marker), thus promoting the neurological recovery after stroke (Geng et al., 2019). EVs from stem cells possess the ability to mimic the beneficial effects of the original cells through the transfer of miRNA and functional proteins (Qiu et al., 2018). Stem cells can participate in the inflammatory response and immune modulation after IS and modulate these pathophysiological changes in an indirect manner by the paracrine effect.

After IS, stem cell-derived EVs can reduce neuroinflammation by turning the microglia into an anti-inflammatory phenotype in rhesus monkeys (Go et al., 2020), which modulates neuroinflammation through interaction with immune recipient cells (Spellicy and Stice, 2020). In addition to microglia inhibition, EVs isolated from MSCs attenuate the activation of astrocytes and T cytotoxic cells, resulting in the inhibition of the expression of pro-inflammatory cytokines such as IL-1, IL-6, and TNF-β (Dabrowska et al., 2019b). After lipopolysaccharide (LPS) stimulation, the administration of stem cell-derived exosomes results in a decrease in astrocyte activation as well as a decrease in expression of TNF-α, IL-1β, and IL-6. These effects suggest that the paracrine effect ameliorates inflammation-induced astrocyte alterations (Xian et al., 2019). The immunomodulatory mechanisms are also established between transplanted stem cells and T cells by paracrine interaction. The paracrine factors released from MSCs suppress CD4^+^–Tbet^+^ (Th1) and CD4^+^–Gata3^+^ (Th2) cells but stimulate CD4^+^–Stat3^+^ (Th17) and CD4^+^–CD25^+^–FoxP3^+^ (Treg) cells (Özdemir et al., 2016). The paracrine effect also has strong immunoregulatory functions contributing to the apoptosis of CD3 (+) T cells when co-cultured with DPSCs (Demircan et al., 2011). The beneficial paracrine effect of stem cell support and protect neurons in ischemic conditions, which is largely attributed to the release of neurotrophic factors such as SDF, NGF, BDNF, GDNF, and FGF (Wakabayashi et al., 2010). The infarct volume in IS rats significantly decreases after MSC transplantation into the infarcted area, accompanied by an increase in bFGF and SDF expression near the infarcted cortex compared with the control group rats (Toyoshima et al., 2015). NSC transplantation can also enhance neurogenesis and activate Akt and Erk1/2 signaling through the release of GDNF after IS (Yuan et al., 2013).

**FIGURE 3:**
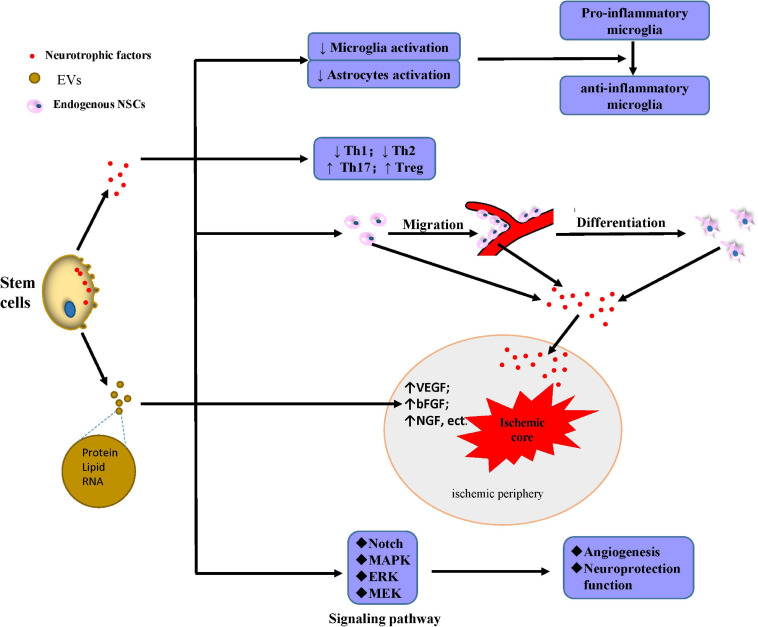
Mechanisms of secretion of protective factors. Stem cells secrete neurotrophic factors or generate EVs mainly containing proteins, lipids, and RNA with multiple neuroprotective effects: inhibiting the activation of microglia and astrocyte, and also promoting microglia shift to the anti-inflammatory phenotype instead of the pro-inflammatory; downregulating Th1 and Th2 and upregulating Th17 and Treg; promoting the migration, differentiation, and secretion of neurotrophic factors to the ischemic periphery; upregulating the content of many factors including VEGF, bFGF, and NGF in the ischemic periphery; and mediating angiogenesis and neuroprotection through the induction of MEK/ERK/MAPK and Notch signaling pathway. Abbreviations: ↑, upregulation; ↓, downregulation; EVs, extracellular vesicles; NSCs, neural stem cells; Th, T helper cells; Treg, regulatory T cells; VEGF, vascular endothelial growth factor; bFGF, basic fibroblast growth factor; NGF, nerve growth factor; MAPK, mitogen-activated protein kinase; ERK, extracellular signal-regulated kinase; MEK, MAP kinase.

Although the neurotrophic factors mentioned above can protect neurons, some miRNAs also exhibit a beneficial function as intercellular messengers. Exosomes from stem cells containing miR-17-92 have significantly robust beneficial effects in the improvement of the neurological function, and they can rescue the boundary area of the infarcted lesion by enhancing neuronal dendrite plasticity and neurite remodeling (Xin et al., 2017). After IS, ADSC-derived exosomes containing miR-126 increase von Willebrand factor and doublecortin (a neuroblast marker) expression, decrease neuronal death, and increase neuron proliferation, improving neuronal functional in IS rats (Geng et al., 2019). Endogenous NSCs and NPCs are located mainly in the dentate gyrus of the hippocampus, the SVZ, and the olfactory bulb and are normally inactive (Obernier and Alvarez-Buylla, 2019); however, the paracrine effect of transplanted cells can promote the proliferation and migration of endogenous NPCs to the injured brain areas (Leal et al., 2017; Geiseler and Morland, 2018). In recent years, interest in the benefits of the paracrine effect of stem cells transplantation has increased the attention on EVs and exosomes due to their low immunogenicity and high BBB permeability and therefore are a potential IS treatment (Figure 3).

### Angiogenesis and Blood–Brain Barrier Repair

Temporary or permanent blood flow deficiency is considered to be the initial cause of a series of pathophysiological changes during IS, and the destruction of BBB directly related to vascular injury is a vital pathological change leading to hemorrhage and poor prognosis (Jiang et al., 2018). Increasing microvessel density and the repair of the damaged BBB to reconstruct the blood flow to the core ischemic area to re-establish the supply of oxygen and nutrients may represent another potentially relevant treatment. Several animal experiments have shown that a variety of stem cells can induce angiogenesis and BBB repair after IS (Uemura et al., 2012; Wang J. et al., 2013; Wang et al., 2015; Zhu et al., 2015; Muir et al., 2020).

Angiogenesis is the formation of new microvessels that branch off from preexisting capillaries (Hatakeyama et al., 2020a). The increased microvessel density and TJ protein (occludin) expression in the border of the animal ischemic core suggest that angiogenesis and BBB repair occur after stem cell treatment (Zacharek et al., 2007). These effects can be divided into two types: cell differentiation and neurotrophic factor secretion. These stem cells directly replace the damaged vascular cells through migration and differentiation after crossing the BBB. Several types of mononuclear cells (MNCs), including CD34^+^, promote angiogenesis through their direct differentiation into smooth muscle cells (SMCs) and ECs in MCAO rats (Terry et al., 2011; Wang J. et al., 2013). In addition to the promotion of angiogenesis, the administration of endothelial progenitor cells (EPCs) seems to be effective in BBB renovation due to their vascular phenotype (Moubarik et al., 2011). The extensive vascular engraftment of human bone marrow EPCs (hBMEPCs) pre-labeled with β-gal suggests a necessary mechanism of direct differentiation for BBB repair (Garbuzova-Davis et al., 2017). MSC differentiation into ECs mediated by the Notch signaling pathway and mitochondrial nanotube transportation are other vital mechanisms of angiogenesis (Zhu et al., 2015). However, the differentiation of grafted stem cells into cells such SMCs or ECs is limited. The existence of direct differentiation has not been confirmed in either HSCs or other stem cell types (Uemura et al., 2012).

The bystander effect exerts a more comprehensive and effective influence in promoting angiogenesis and BBB preservation. Coordinated remodeling of ECs, basal matrix, and pericytes induces angiogenesis to produce new blood vessels (Li and Carmeliet, 2018). This complex process is regulated by a variety of cytokines and receptors; and some mediators such as VEGF and TGF-β modulate angiogenesis during the natural pathophysiological process (Kanazawa et al., 2019). VEGF, which has been widely studied in the transplantation of MNCs and MSCs, is able to promote angiogenesis by the bystander effect. The expression of other trophic factors such as BDNF, GDNF, Angiotensin 1 (Ang-1), Angiotensin 2 (Ang-2), IGF-1, and bFGF is upregulated after MNC treatment, contributing to the formation of immature vessels (Ikegame et al., 2011; Li et al., 2020b). Most of these cytokines promote angiogenesis by binding to specific receptors. For example, VEGF binding to VEGF receptor-2 (VEGFR-2) with high tyrosine-kinase activity improves angiogenesis, while binding to another receptor such as VEGFR-1 results in no protective effect. Some studies have shown that VEGFR-2 increases with VEGF upregulation after stem cell therapy in ischemic rats and that the same pattern was also observed in Ang-1 and its receptor Tie2 (Zacharek et al., 2007; Toyama et al., 2009). The angiogenic effect of VEGF in IS also varies according to the time window, source, and administration mode. Early administration of recombinant human VEGF in ischemic rats can significantly aggravate BBB leakage and hemorrhagic transformation (Zhang et al., 2000). The regulation of nutritional factors in the molecular mechanism of angiogenesis is multifaceted and often associated with neurogenesis (Hatakeyama et al., 2020a). A recent study found that autophagy may be a cause of vascular injury and that EVs secreted from MSCs can activate signal transduction and the activation of the transcription factor STAT3 to inhibit the autophagy-related pathway (Xia et al., 2020).

There has been a recent increase in miRNA research, and EVs containing certain miRNAs produce protective effects in stem cells through the activation of signaling pathways related to angiogenesis and neurogenesis (Bang and Kim, 2019; Moon et al., 2019). ADSCs *in vitro* secrete miRNA-181b, which promotes the angiogenesis in brain microvascular ECs through the transient receptor potential melastatin 7 (TRPM7) axis (Yang et al., 2018). MiRNAs can also regulate stem cell function; for example, miR-126 overexpression can promote the proliferation, migration, and tube formation abilities of EPCs and also the increase NO production of EPCs via activation of the PI3K/Akt/eNOS pathway, thus increasing cerebral microvascular density and angiogenesis (Pan et al., 2018). Angiogenesis is considered to be helpful for neurogenesis and the formation of neurovascular units and is favorable for regeneration and functional recovery of neurons.

### Neural Circuit Reconstruction

Cells with different properties and functions in the brain form neural circuits and neural networks at different levels through various complex connections; and positive and negative feedback regulates the complex functions of the brain (Helmstaedter, 2013). IS results in the loss of brain neurons and the destruction of nerve connections, leading to severe neurological dysfunctions. Adult mammals have limited brain regeneration abilities (Li et al., 2020b), meaning that stem cell therapy can play an important role in the repair and functional reconstruction of damaged neural circuits.

Neurogenesis involves the generation of new functional neurons from NSCs, which includes their proliferation, migration, and differentiation into mature neurons. In the adult brain, endogenous neurogenesis continuously persists in two areas, the SVZ of the lateral ventricle and the subgranular zone (SGZ) of the hippocampus; and it increases after IS (Hatakeyama et al., 2020b). Several signaling pathways and extracellular factors are involved in the regulation of neurogenesis, which is further enhanced by stem cell treatment (Hatakeyama et al., 2020a,b). The activation of NSCs proliferation is regulated by Notch-mediated signaling and other transcription factors such as Wnt and the Sonic Hedgehog signaling pathway (Faigle and Song, 2013). The proliferation of endogenous NSCs can be enhanced by several extracellular factors such as FGF-2, IGF-1, and platelet-derived growth factor (PDGF) (Hatakeyama et al., 2020a), all of which are abundantly secreted by transplanted stem cells. Following NSC proliferation, several mechanisms mediated by the administered stem cells promote neuroblast migration from the SVZ to the peri-infarcted region, especially where angiogenesis occurs (Ruan et al., 2015). BDNF secreted by ECs promotes vascular-guided NSC migration (Grade et al., 2013). In newborn capillaries working as a scaffold, β1 integrin expressed in neuroblasts can adhere to laminin expressed in the vessels to achieve migration (Grade et al., 2013). MSC treatment promotes NSC migration after the release of neurotrophic factors beneficial to angiogenesis. After proliferation and migration, NSCs differentiate into different mature neurons according to the local microenvironment in which they are located to repair damaged brain areas (Kawabori et al., 2020a). Angiogenesis plays a crucial role in the differentiation of NSCs into mature neurons in the ischemic area, as observed in *in vitro* experiments (Shen et al., 2004) and also in a recent animal study reporting that angiogenesis in the cortex can induce NSC transition from a proliferative form to a differentiated one (Shen et al., 2004). Transplanted stem cells can also perform neurogenesis by directly differentiating into neuronal cells. More than 50% of transplanted stem cells in the ischemic brain express a neuronal phenotype at 2 months after cell transplantation (Kawabori et al., 2012, 2013).

The sprouting of axons increases the connections between different areas of the brain; however, in the adult brain after IS, axonal sprouting is limited by the failure of neurogenesis to be fully activated and by inhibitory cytokines (Carmichael et al., 2017). Treatment with stem cells such as MSCs, NSCs, and bone marrow-derived stem cells (BMCs) ameliorates this defect by promoting angiogenesis and bystander effects (Neuhuber et al., 2005; Fujioka et al., 2017; Huang et al., 2019). VEGF released from vessels, and laminin/β1 integrin signal in ECs induced by microtubule assembly and stabilization are both crucial aspects in promoting axonal outgrowth (Jin et al., 2006; Fujioka et al., 2017). Since axonal sprouting is limited before angiogenesis, this suggests a close connection between axonal sprouting and angiogenesis (Kanazawa et al., 2017a). Treatment with BDNF, gene-transfected MSCs, and axonal outgrowth can also be promoted (Hira et al., 2018).

Synaptogenesis is a long back-and-forth process involving synapse formation, synapse stabilization, and activity-dependent synapse refinement and elimination, all important aspects to maintain the stability and precision of neural circuits (Cepeda et al., 2002). Research on synaptogenesis after stem cell therapy is currently focused on the changes in characteristics. Following treatment with reprogrammed human neural precursor cells derived from iPSCs, the increased expression of the presynaptic vesicle protein synaptophysin enhances synaptogenesis after IS (Vonderwalde et al., 2020). Another study demonstrated that stem cell factor and G-CSF may promote synaptogenesis through VEGF-α (Ping et al., 2019).

**FIGURE 4:**
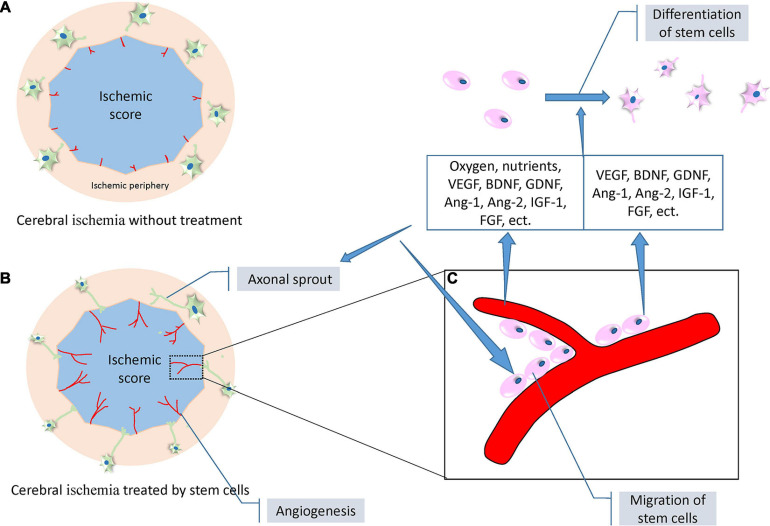
A set of diagrams of angiogenesis, neurogenesis, and axonal outgrowth under the treatment of stem cells following ischemic stroke (modified from the reference of Masato Kanazawa et al., 2019). **(A)** A proposed diagram depicting the location of angiogenesis and axonal outgrowth after ischemic stroke without intervention. Weak angiogenesis (red lines) occurs at the border of the ischemic core (blue) and peripheral areas, and limited axonal sprouting occurs in the neurons (green cells) of the peripheral areas. **(B)** A proposed diagram depicting enhanced angiogenesis and evident axonal sprouting and outgrowth under the treatment of stem cells. **(C)** A representative diagram expanded from a certain area of the diagram in (**B**). Stem cells (pink) adhering to the newly formed blood vessels to achieve migration. The blood vessels and stem cells themselves secrete a variety of neurotrophic factors including VEGF, BDNF, GDNF, Ang-1, Ang-2, IGF-1, and FGF. In addition, blood vessels release additional oxygen and nutrients to promote the proliferation, migration, and differentiation of stem cells, and axonal outgrowth. Abbreviations: VEGF, vascular endothelial growth factor; BDNF, brain-derived neurotrophic factor; GDNF, glial cell-derived neurotrophic factor; Ang-1, angiotensin 1; Ang-2, angiotensin 2; IGF-1, insulin-like growth factor 1; FGF, fibroblast growth factor.

Stem cell therapy mechanisms can be categorized into two distinct forms: direct action characterized by cell migrating to the infarct area and bystander effort, and indirect action characterized by improving local microenvironment through angiogenesis, neurogenesis, and the regulation of inflammation. Both mechanisms can combine to improve neuronal function after IS; however, the different mechanisms do not receive the same level of focus in the current research. There has been a lot of fundamental research into exosomes and their related secreted factors, as a medium for information exchange between cells, in stem cell therapy. However, there is limited research on direct differentiation of stem cells, which may be related to the current lack of effective detection methods. The examination of new key molecules or regulatory pathways that regulate indirect action should also be encouraged, as these mechanisms are part of a closely connected network. For example, neuroinflammation reduction can be assisted by the alleviation of inflammatory factor infiltration when the BBB is repaired, and the regulation of inflammation provides a favorable microenvironment for angiogenesis or neurogenesis, which in turn supplies oxygen and nutrients to ischemic tissue and neurotrophic factor necessary for axonal outgrowth. Stem cell therapy, through the regulation of multiple mechanisms, is an innovative treatment with outstanding advantages (Figure 4).

## Clinical Stem Cell Therapy Trials

Over the last decades, a large number of preclinical studies have been carried out on the role of stem cell therapy in IS treatment. Thanks to the promising results of the preclinical studies, an increasing number of clinical trials have investigated the safety and efficacy of different stem cells in IS treatment. Some issues still need to be resolved, such as the choice of the cell type, cell dose, time of transplantation, and injection routes, all of which benefit from a close link between basic and clinical research (Table 1).

**TABLE 1:** Summary of stem cells administered in clinical trials.

Stem cell types	Routes	Timing	Dose	Outcome	References
MSCs	Intracerebral	Subacute phase	2.5–10 × 10^6^	No adverse events, and dose-limiting toxicity or death and improved clinical outcomes.	Steinberg et al., 2018
MSCs	Intravenous	Subacute phase	0.6–1.5 × 10^8^	Reduced lesion volume as demonstrated by MRI and functional recovery with no tumor or abnormal cell growth.	Honmou et al., 2011
MSCs	Intravenous	Chronic phase	1.5 × 10^6^/kg	Safety and improvements were seen in the National Institutes of Health Stroke Scale, Barthel Index, Mini-Mental Status Exam, and Geriatric Depression Scale scores during the follow-up study.	Levy et al., 2019
MSCs	Intravenous	Acute phase	1 × 10^6^/kg	No adverse events, neurologic and systemic complications, and tumor development and improved neurologic recovery with decreased infarct size.	Díez-Tejedor et al., 2014
MSCs	Intravenous	Subacute phase	2.875 × 10^9^	No significant difference between the BMSC arm and control arm in the Barthel Index score, Modified Rankin Scale shift analysis, National Institutes of Health Stroke Scale, and infarct volume.	Prasad et al., 2014
HSCs	Intra-arterial	Acute phase	1 × 10^8^	Improvements in the Modified Rankin Scale score and National Institutes of Health Stroke Scale score and reductions in lesion volume during a 6-month follow-up period.	Banerjee et al., 2014
HSCs	Intrathecal	Chronic phase	0.8–3.3 × 10^7^	Improved muscle tone, rigidity, and motor power.	Wang L. et al., 2013
NSCs	Intracerebral	Chronic phase	2 × 10^6^/5 × 10^6^	No immunological or cell-related adverse events, decreased infarct lesions observed by MRI.	Kalladka et al., 2016
NSCs	Intracerebral	Subacute to chronic phases	2 × 10^7^	4 of 23 participants improved by the pre-specified Action Research Arm Test subtest level; transient procedural adverse effects were observed, but no stem cell-related adverse events.	Muir et al., 2020
DPSCs	Intracerebral	Chronic phase	1 × 10^7^	Safe, feasible, and observed function recovery.	Nagpal et al., 2016
PBSCs	Intracerebral	Chronic phase	3–8 × 10^6^	No serious adverse events; improvements in the National Institutes of Health Stroke Scale, Modified Rankin Scale, European Stroke Scale (ESS), and ESS Motor Subscale from the baseline to the end of the 12-month follow-up period.	Chen D.C. et al., 2014
MSCs	Intravenous	Subacute phase	1 × 10^8^	No adverse cell-related, serological, or imaging-defined effects, and the Barthel index and Modified Rankin Scale score consistently improved during the follow-up period with less prominent infarcted areas.	Bang et al., 2005a
BMMNCs	Intra-arterial	Subacute phase	5 × 10^8^	No procedure-related mortality, complications, new infarct, or symptomatic intracranial hemorrhage and good clinical outcome observed by Modified Rankin Scale score.	Bhatia et al., 2018
BMMNCs	Intravenous	Acute phase	2.5 × 10^8^/3.4 × 10^8^	Improved cerebral blood flow and metabolic rate of oxygen consumption 6 months after treatment with significantly better neurologic outcomes.	Taguchi et al., 2015
BMMNCs	Intravenous	Acute phase	1 × 10^7^/kg	No severe adverse events and diffusion tensor tractography illustrates an increase in cortical spinal tracts fiber volume.	Vahidy et al., 2019
Fetus neuronal cell	Intracerebral	Chronic phase	5 × 10^6^	Safe and feasible but no evidence of significant benefit in motor function were observed.	Kondziolka et al., 2005
Fetal porcine cells	Intracerebral	Subacute to chronic phase	1 × 10^6^	Temporary worsening of motor deficits and seizure; 2 of 5 patients showed improvement in speech, language, and/or motor impairments over several months and persisted at 4 years. The study was terminated by the Food and Drug Administration.	Savitz et al., 2005

***Abbreviations:** PBSCs, peripheral blood stem cells; MMCs, marrow-derived mononuclear cells; BMMNCs, bone marrow mononuclear cells; MSCs, mesenchymal stem cells; HSCs, hematopoietic stem cells; DPSCs, dental pulp stem cells; NSCs, neural stem cells.*

### Choice of Stem Cell Types

Several types of stem cells have been widely studied in IS animal models, and some of them have been examined in clinical trials. All the stem cells used play important roles in the treatment of IS through different mechanisms. Stem cells can be obtained from different tissues of the same IS patient, including bone marrow, adipose tissue, dental pulp, and connective tissue; or they can be commercially purchased. Although several clinical studies have investigated the safety and effectiveness of some stem cells, more attention has focused on BMCs such as MSCs (Steinberg et al., 2018). Tissue-derived MSCs remain the main cells used in stem cell therapy to treat IS because of their easy access and different protective mechanisms; however, the choice of a proper stem cell type in clinical IS treatment should take into account not only the advantages mentioned above but also the accessibility, ethical issues, risk of posttransplant rejection, allergies, and even tumorigenicity. When there is a high risk of posttransplant rejection, autologous stem cells are more suitable (Bang et al., 2005a; Chen D.C. et al., 2014; Trounson and McDonald, 2015). However, since IS is an acute disease, the time required to culture some autologous stem cells, such as BM-MSCs to reach the number required for transplantation, limits their application in acute phase of IS, and allogeneic stem cells are therefore usually selected. In a phase I safety study, the fourth injection of allogeneic human UCB cells proved to be safe in adults with IS, and patient recovery was improved (Laskowitz et al., 2018). Although allogeneic stem cells have promising prospects, some issues still need to be solved, including the lack of systems guaranteeing the standard quality of stem cells administered in clinical trials or treatments, and the expense of producing, preserving, and transferring these cells. Although most methods used for stem cell therapy appear to be safe, some studies have reported on the continuous limitations in the administration of some stem cells such as ESC and iPSCs because of the risk of tumorigenicity, immunogenicity, and heterogeneity (Yamanaka, 2020). The transplantation of iPSCs can increase the risk of teratoma, especially in IS models (Yamashita et al., 2011), suggesting that the safety of stem cells should be evaluated. On the other hand, several studies have obtained encouraging results using ESC and iPSC; thus, the confidence of using them in clinical treatments is increased. The organ-on-a-chip model, which is a sensitive *in vitro* system, is being developed to effectively predict the possibility of tumor formation (Sato et al., 2019). There are many mechanisms characterizing the underlying therapeutic effect of stem cells, meaning that one of them may be making the main contribution. For example, the bystander effect in MSCs after IS can effectively improve functional recovery, NSCs possess a strong ability to differentiate into neurons and reconstruct the neural circuit, and HSCs can significantly stimulate angiogenesis, which ameliorates OGD damage. The self-renewal and differentiation abilities of stem cells greatly differ. During IS, the pathophysiological changes after stroke are due to a dynamic process, which in turn requires a flexibility in stem cell choice dependent on individual patient’s conditions. In the acute phase of IS, the main danger can be the acute cascade of neuroinflammatory factors, damage of the BBB, and the apoptosis of CNS cells, while sustained neuroinflammation, the formation of glial scar, and the destruction of the neural circuit influence the functional recovery in the subacute to chronic phase. MSC administration can significantly reduce the volume of the ischemia lesion and increase the functional recovery with the improvement of the outcome of the Modified Rankin Scale (mRS) and National Institutes of Health Stroke Scale (NIHSS) (Honmou et al., 2011; Díez-Tejedor et al., 2014). The paracrine effect of MSCs contributes, at least in part, to this neuroprotective effect. The safety of HSCs therapy is proven (Wang L. et al., 2013), and it can significantly improve the functional outcome from the baseline to the end of the 12-month follow-up period (Chen D.C. et al., 2014). Based on the different features of stem cell types and the pathophysiological change in each phase of IS, it is advisable that the stem cell therapeutic strategy should be adjusted to the pathophysiology features according to further clinical studies.

### Stem Cell Administration Routes

Different administration routes including intravenous, intra-arterial, intrathecal, intracerebroventricular, intracerebral, subarachnoid, intranasal, and intraperitoneal routes have been used in preclinical trials, and many clinical studies have reported on the safety of these administration routes. The intravenous and intracerebral routes are widely used in published clinical studies; however, it is not clear which of them represents the optimal route.

The intra-arterial route is less invasive than the others. Like the intravenous route, the intra-arterial route can be used with a large number of cells (Bang et al., 2005b; Savitz et al., 2011b); however, the advantage of the intra-arterial route is that stem cells can bypass the peripheral filtering organs such as the liver, the spleen, and the lungs, leading to a higher cell engraftment into the brain and biological distribution (Berkhemer et al., 2015). A study using stem cells transduced with a firefly luciferase reporter gene for bioluminescence imaging (BLI) revealed that the BLI signal in the brain of the rats treated with the intra-arterial route is significantly higher than that in the lungs and liver and is higher than the signal in the brain after intravenous injection (Pendharkar et al., 2010). The MT is widely used at 3–4.5 h after IS with the help of a catheter (Wardlaw et al., 2014), and it is worth considering the combination of this and the intra-arterial route for the delivery of stem cells into the ischemic lesion. However, there is still the risk that the stem cells can stick together and form microemboli, blocking the vasculature and leading to occlusions and local hypoperfusion (Walczak et al., 2008). The intravenous route and intra-arterial route show the same protective features or feasibility, but the intravenous route is the one mainly used in recently published studies because of repeatability, lower invasiveness, easy access, and inessentiality of special equipment such as catheters. The intravenous route greatly contributes to the amelioration of neuroinflammation, activation of immune cells, and neuron apoptosis thanks to the stem cells’ secretion of neurotrophic factors to modulate neuroinflammation or immune response. The transplanted stem cells administered by intravenous injection go over the pulmonary vascular system arriving in the infarcted area in IS (Misra et al., 2012); however, a large proportion of transplanted stem cells are trapped in the peripheral organs (Kawabori et al., 2012; Boltze et al., 2015). Interestingly, the stem cells trapped in the spleen improve the splenic inflammatory response, reduce neuron apoptosis in the brain, and increase the functional recovery (Vasconcelos-dos-Santos et al., 2012; Huang et al., 2013; Acosta et al., 2015). Like the stem cells transplanted by the intra-arterial routes, stem cells transplanted by the intravenous one can also lead to the formation of microemboli causing fatal pulmonary embolism. Nevertheless, the intravascular routes (intravenous and intra-arterial route) mainly contribute to the functional recovery that may mainly rely on the bystander effect, while the intrathecal, intracerebroventricular, intracerebral, and subarachnoid routes are invasive routes that can directly deliver the stem cell into the CNS, thus facilitating the reconstruction of the neural circuit and the replacement of the damaged brain tissues in IS (Kelly et al., 2004). After IS, stem cell transplantation through the intracerebral route resulted in more transplanted stem cells appearing near the infarcted lesions because of the direct injection of millions of stem cells under the help of invasive surgery. Approximately 1/3 can migrate to the ischemic area (Darsalia et al., 2007; De Feo et al., 2012), thus achieving the highest stem cell engraftment of all administration routes. However, invasive surgery can bring a second attack to the patient, and intracerebral routes are predominantly used in the chronic phase of IS, according to published clinical studies (Kondziolka et al., 2005; Chen et al., 2013; Steinberg et al., 2016; Steinberg et al., 2018). The amount of transplanted stem cells is limited to avoid the mass effect, and the harsh microenvironment containing inflammatory factors can influence the grafted stem cells survival, differentiation, proliferation, and migration (Bühnemann et al., 2006). The risk of additional brain damage caused by injection needles and invasive surgery-related complications including death, seizure, and infection should be taken into consideration (Jeong et al., 2014; Kawabori et al., 2020b). The intracerebroventricular route may be less invasive than the intracerebral route, but complications such as hydrocephalus and liquorrhea may occur after administration. Recently, a novel delivery route for stem cells for IS therapy has been reported and gained attention because of its effectiveness and minimal invasiveness. Indeed, the intranasal administration of cells allows the bypass of the BBB and increases the migration into the lesions (Wei et al., 2013, 2015), and MSCs administered through this route show great potential to enhance neurovascular regeneration and improve functional recovery (Wei et al., 2015). However, the intranasal and intraperitoneal routes are still at the stage of preclinical studies and need further safety and efficacy evaluation before use in large-scale clinical trials. So far, the strong connection between routes of administration and the target of stem cell therapy is quite evident. In the acute phase of IS, the neuroinflammation and neural cell death greatly contribute to the ischemia damage to the CNS, and at present, the intravascular routes are preferable in order to use the bystander effect. However, in the chronic phase of IS, when the acute pathophysiology changes are stabilized, the replacement of the damaged tissue can be more important, and intracerebral or other invasive routes are more proper to directly enhance cellular intercommunication. Nevertheless, many issues still exist, which should be taken into consideration when deciding the optimal choice of administration routes.

### Time of Administration

Nowadays, not all patients are eligible for reperfusion treatment because of the narrow time window, although MT and the administration of tPA are widely used in clinical practice. In the different phases of IS such as acute, subacute, and chronic phases, many pathophysiological changes occur. However, some particular pathophysiological changes play major roles in different phases, enabling us to consider specific treatments. Based on the pathophysiology features of different IS phases, treatment strategies regarding the administration time have been discussed in clinical and preclinical studies. In acute or subacute phases of IS, a series of cytokines, chemokines, and reactive oxygen species are released, which change the microenvironment, finally damaging the neural cells (Choi, 1992; Adibhatla and Hatcher, 2010; Dabrowska et al., 2019a; Jayaraj et al., 2019), although some of these factors have positive effects. For example, the release of some chemokines including SDF after the onset of IS can induce the migration of exogenous transplanted stem cells (Chen Y. et al., 2014). Administration of stem cells in the early phases of IS can reduce neuroinflammation, modulate the immune system, regulate the microenvironment, and rescue neurons and glial cells in the peri-ischemia areas (Bang, 2016), suggesting that the focus of cell therapy in the acute and subacute phases should be on the rescue of ischemia-damaged cells (Savitz et al., 2011a). In the rat IS model, the administration of NSCs shortly after stroke (48 h) results in a better stem cell survival compared with stem cells administered 6 weeks after stroke. The same study also reported that the activation of microglia after the onset of IS reaches the maximum level at 1 to 6 weeks after stroke (Darsalia et al., 2011), suggesting that the administration of stem cells before the total establishment of neuroinflammation increases the survival of stem cells and improves their therapeutic effect. Thus, according to the instruction of preclinical studies, some clinical trials reported the safety and efficacy of stem cell administration in an early phase of IS (Kim et al., 2013; Shichinohe et al., 2017; Bhatia et al., 2018), resulting in a good clinical outcome, since 80% of the patients who received stem cell administration in the early phase of IS show good functional recovery (Bhatia et al., 2018). The early phase of IS is the appropriate period for traditional reperfusion treatment and stem cell therapy as well, but functional recovery in this phase is influenced by many factors including the spontaneous recovery of endogenous angiogenesis or neurogenesis. It is therefore important to take these aspects into consideration when assessing the effect of stem cell therapy in future clinical studies. The purpose of stem cell therapy in the chronic phase is totally different because it focuses mainly on neurogenesis, angiogenesis, and synaptic plasticity in peri-ischemic areas to restore neuronal functions. Trials in this phase mainly transplant the stem cells into peri-infarcted areas, promoting intercommunication between exogenous stem cells and cells that survived in the CNS, which increases the reconstruction of the neural circuit (Kalladka et al., 2016). Some clinical trials have reported the efficacy of stem cell therapy in the chronic phase of IS by intracerebral and intravascular routes (Chen D.C. et al., 2014; Levy et al., 2019; Muir et al., 2020). However, the study was interrupted because of a temporary worsening of the motor deficits and seizures when stem cells were administered in the chronic phase (Savitz et al., 2005). Therefore, the limited efficiency to replace the damaged tissue in the chronic phase of IS should be taken into consideration because of the formation of glial scar, irremediable loss of amount of brain parenchyma, and lack of blood supply to the transplanted stem cells (Colton, 1995; Diotel et al., 2020). Despite these negative aspects, tissue engineering hopes to solve the problems of administering stem cells in the chronic phase of IS. Some artificial scaffolds and matrix can serve as a platform to provide niches that can increase survival, proliferation, and functions of the transplanted stem cells (Murphy et al., 2010; Gopalakrishnan et al., 2019), thus solving the problems mentioned above, although the effect of stem cell therapy in the chronic phase of stroke still needs more research.

### Cell Dose

The stem cell dose is flexible but should be adapted to the particular cell type or transplantation route used. The cell dose applied in clinical trials can widely differ from 10^6^ to 10^9^. Considering the easy access and the lowest invasiveness of the intravenous or intra-arterial routes, the cell dose can reach more than 10^8^ (Taguchi et al., 2015; Hess et al., 2017; Bhatia et al., 2018; Levy et al., 2019; Vahidy et al., 2019). For example, the number of transplanted stem cells by intravenous route can reach 1,200 million, with no dose-limiting toxicity events, allergic reactions, and treatment-emergent adverse events, revealing the safety of such cell dose (Hess et al., 2017). A recent study used two different cell doses (10^5^ and 10^6^) of MSCs that were intravenously administered. Although both doses improved functional recovery, the result revealed higher stem cell engraftment in the peri-infarct area at the higher dose, suggesting that the therapeutic effect of stem cells is positively correlated with the cell dose in a proper range (Kawabori et al., 2013). A meta-analysis regarding the relationship between the therapeutic effect and dose of transplanted stem cells did not show a significant linear regression relationship between them (Wang et al., 2016). Since a higher stem cell number can lead to the mass effect when administered by intracerebral route, the transplantation dose should be limited to no more than 10^7^ (Kalladka et al., 2016, 2019; Nagpal et al., 2016; Muir et al., 2020). A heterogeneous functional outcome is still observed under the same cell dose administered by the intracerebral route, probably because of patients in different conditions and the influence of a particular microenvironment. Some studies on material technology give hope on the use of several engineered matrix such as a hyaluronic acid (HA)-based self-polymerizing hydrogel that can serve as a platform for transplanted stem cells, miming the native neural tissue environment, and minimizing the influence from the environment of the peri-ischemia area after transplantation into the stroke core. This improves the survival and migration of stem cells, finally differentially modulating the transplanted cell fate (Moshayedi et al., 2016; Gopalakrishnan et al., 2019). Administration of the engineered matrix can significantly increase the implantation efficiency and can influence the transplantation dose as well. Only few studies are available discussing the appropriate cell dose in stem cell therapy and considering the risk of formation of tumors and thrombosis, and thus, further studies should investigate these issues before the widespread use of stem cell therapy in clinical practice.

## Conclusion

Our review summarizes the mechanisms of stem cell therapy in detail, as well as several key parameters elucidated by the current clinical research, providing a reference and a guide in the direction of further research. Stem cell therapy is a promising treatment strategy to cure IS, with multi-mechanisms that can effectively cope with the pathophysiological changes after stroke. In addition, many preclinical and clinical trials have revealed promising prospects in the administration of stem cell therapy to cure IS. However, much research still needs to be performed, and new discoveries may help develop a better use or modification of stem cell therapy or the treatment methods derived from it. Current clinical trials are still mainly in phase I of safety. The next step may require exploration of more clinically suited issues, such as the choice of stem cell types, the route of stem cell administration, the administration time, and the cell dose. These still unclear aspects require results from double-blind studies with larger cohorts.

## Author Contributions

All the authors participated in analyzing and discussing the literature, commenting on and approving the manuscript. AS and LW supervised the research, led the discussion, and wrote and revised the manuscript. All authors read and approved the final manuscript.

## Conflict of Interest

The authors declare that the research was conducted in the absence of any commercial or financial relationships that could be construed as a potential conflict of interest.
